# Supplementary material for: Carbon Dot Emission Enhancement in Covalent Complexes with Plasmonic Metal Nanoparticles
Source: Nanomaterials (Basel). 2023 Jan 4;13(2):223. doi: 10.3390/nano13020223 (PMC9867019; doi:10.3390/nano13020223)
Supplement: Supplementary file 1 [file nanomaterials-13-00223-s001.zip › nanomaterials-2127104-supplementary.pdf]

Carbon Dot Emission Enhancement in Covalent Complexes with Plasmonic Metal Nanoparticles

Irina A. Arefina [1], Danil A. Kurshanov [1], Anna A. Vedernikova [1], Denis V. Danilov [2], Aleksandra V. Koroleva [3], Evgeniy V. Zhizhin [3], Aleksandr A. Sergeev [4], Anatoly V. Fedorov [1], Elena V. Ushakova [1,*] and Andrey L. Rogach[5]

[1] International Research and Education Centre for Physics of Nanostructures, ITMO University, Saint Petersburg 197101, Russia

[2] Interdisciplinary Resource Centre for Nanotechnology, Saint Petersburg State University, Saint Petersburg 199034, Russia

[3] Centre for Physical Methods of Surface Investigation, Saint Petersburg State University, Saint Petersburg 199034, Russia

[4] Department of Physics, Hong Kong University of Science and Technology, Hong Kong S.A.R. 999077, China

[5] Department of Materials Science and Engineering, and Centre for Functional Photonics (CFP), City University of Hong Kong, Hong Kong S.A.R. 999077, China

[*] Correspondence: elena.ushakova@itmo.ru

1. Syntheses of CDs.

Synthesis of CD-1. 1.05 g of citric acid and 335 μL of ethylenediamine were dissolved in 10 mL of water. The solution was heated in a Teflon-lined autoclave at 160 °C for 6 h. After cooling down to room temperature, the suspension was dialyzed against deionized water using 14 kDa dialysis bag for 1 day. After dialysis, the mass concentration of the CD stock solution was determined to be ∼25 mg/mL.

Synthesis of CD-2. 2 g of urea was mixed with 1 g of citric acid and dissolved in 10 mL DMF. The solution was heated in a Teflon-lined autoclave at 160°C for 6 h. After cooling down to room temperature, the suspension was dialyzed against deionized water using 14 kDa dialysis bag for 1 day. The mass concentration of the CD stock solution was determined to be ∼30 mg/mL.

Synthesis of CD-3. 0.6 g of o-phenylenediamine and 0.5617 g of benzoic acid were dissolved in 25 mL of ethanol. The solution was transferred into a Teflon-lined autoclave and heated at 160°C for 6 h. The resulting solution was purified from large particles and agglomerates by filtration through a 0.22 μm membrane, and dialyzed against deionized water using 6-8 kDa dialysis bag for 2 days. The mass concentration of the CD stock solution was determined to be ~44.5 mg/mL.

2. Syntheses of MNPs.

Synthesis of Au-PEG NPs. 10 mL of water solution containing 0.11 g (0.3 mmol) of PEG was vigorously mixed with 0.118 g (0.3 mmol) of $HAuCl_4 \times 3H_2O$ dissolved in 30 mL of H_2O for 10–15 min at room temperature. Thereafter, 30 mL of the freshly prepared aqueous solution of $NaBH_4$ (0.113 g, 3 mmol) was added to the mixture and left stirring overnight.

Synthesis of Au-PEG NPs. 10 mL of water solution containing 0.11 g (0.3 mmol) of PEG was vigorously mixed with 0.118 g (0.3 mmol) of $HAuCl_4 \times 3H_2O$ dissolved in 30 mL of H_2O for 10–15 min at room temperature. Thereafter, 30 mL of the freshly prepared aqueous solution of $NaBH_4$ (0.113 g, 3 mmol) was added to the mixture and left stirring overnight.

Synthesis of AgNPs. 500 μL of cysteamine (1.4 M) and 500 μL of $AgNO_3$ (0.234 M) were sequentially added to 20 mL of DMF under stirring in an ice bath. After that, 3 mL of fresh cold $NaBH_4$ (0.21 M) was added dropwise. The reaction mixture was stirred for 20 min, after which AgNPs were centrifuged for 10 min at 10 000 rpm and the precipitate was dispersed in water. The obtained AgNPs with an estimated mass concentration of 1.104 mg/mL were further stored in a dark place at 2-5°C. The mass concentration was calculated using the average particle size determined by TEM, according to the equation $C = \frac{N_{total}}{NVN_A}, N = \frac{79}{100}\frac{\pi D^3}{6}\frac{1}{0.0125}$ from reference [1], where N is the average number of silver atoms, D is the average diameters of the particles, N_{total} - the total number of silver atoms, V is the volume of solution, and N_A is the Avogadro's constant. The total number of silver atoms was calculated by considering their volume ratio of 79% in the cubic structure. Since the radius of a silver atom is 0.144 nm, its volume is 0.0125 nm^3.

Synthesis of AuNPs. 400 μL of cysteamine (213 mM) and 40 mL of $HAuCl_4$ (1.42 mM) solution were mixed under gentle stirring in a dark place. After that 10 μL of fresh cold $NaBH_4$ (10 mM) was added under vigorous stirring for 10 min, followed by a slower stirring for 30 min. Cysteine stabilized AuNPs with an estimated mass concentration of 0.277 mg/mL were stored in a dark place at 2-5 °C. The mass concentration was calculated using the average particle size determined by TEM, according to the equation $C = \frac{N_{total}}{NVN_A}, N = \frac{\pi}{6}\frac{\rho D^3}{M}$ from reference [2], where N is the average number of gold atoms, ρ is the density for fcc

gold, M - atomic weight of gold, D is the average diameters of the particles, N_{total} - the total number of gold atoms, V is the volume of solution and N_A is the Avogadro's constant.

References:

1. Liu, X.; Atwater, M.; Wang, J.; Huo, Q. Extinction Coefficient of Gold Nanoparticles with Different Sizes and Different Capping Ligands. *Colloids Surf. B Biointerfaces* **2007**, *58*, 3–7, doi:10.1016/J.COLSURFB.2006.08.005.
2. Wimuktiwan, P.; Shiowatana, J.; Siripinyanond, A. Investigation of Silver Nanoparticles and Plasma Protein Association Using Flow Field-Flow Fractionation Coupled with Inductively Coupled Plasma Mass Spectrometry (FlFFF-ICP-MS). *J. Anal. At. Spectrom.* **2014**, *30*, 245–253, doi:10.1039/C4JA00225C.

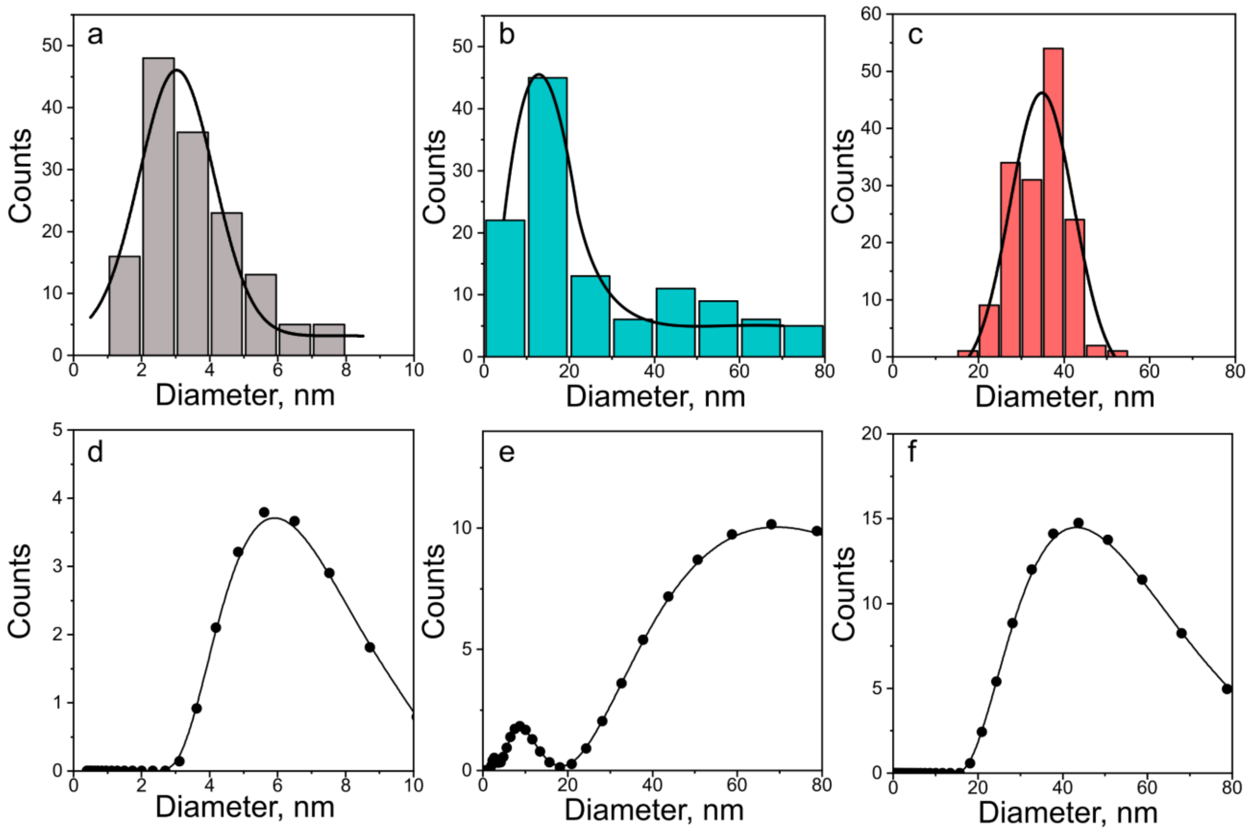

Figure S1. Size distributions of (a, d) CD-3, (b, e) AgNPs, and (c, f) AuNPs determined by TEM (a-c) and DLS (d-f).

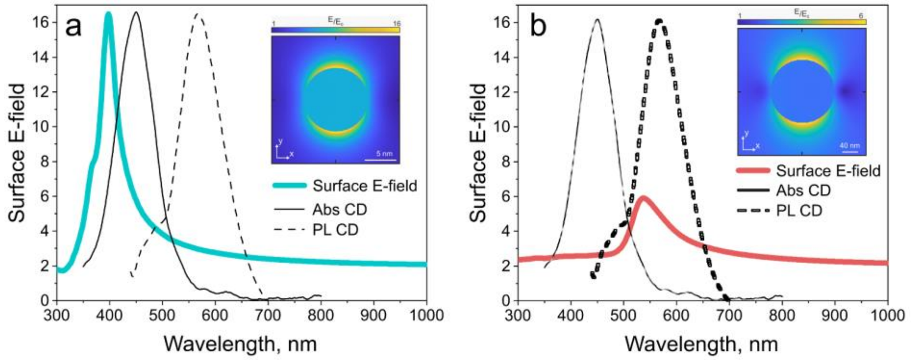

Figure S2. Numerically simulated electric field enhancement (E/E_0) at the surface of (a) 9-nm Ag NPs (cyan line) and (b) 45-nm Au NPs (red line), plotted together with the normalized absorption (black solid lines) and PL (black dashed lines) spectra of CD-3. Insets shows the electric field enhancements (E/E_0) around the respective single MNP at the wavelength of scattering resonance.

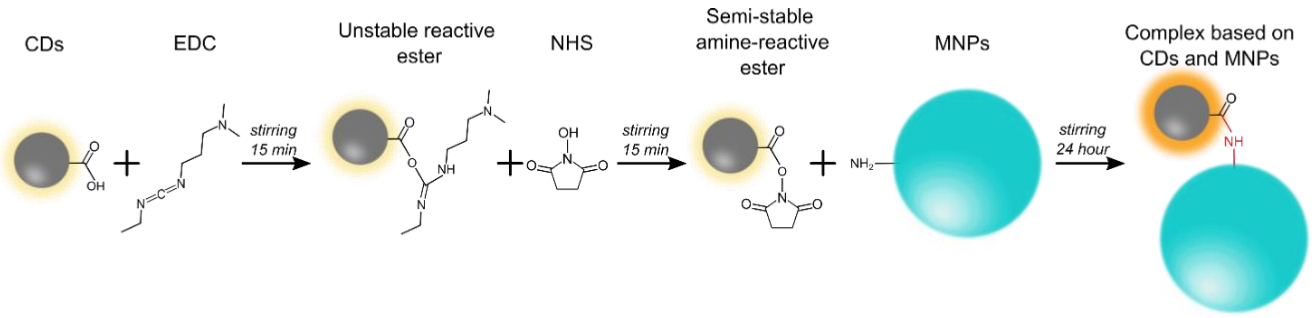

Figure S3. Conjugation strategy leading to the formation of covalent complex between CD-3 and MNPs.

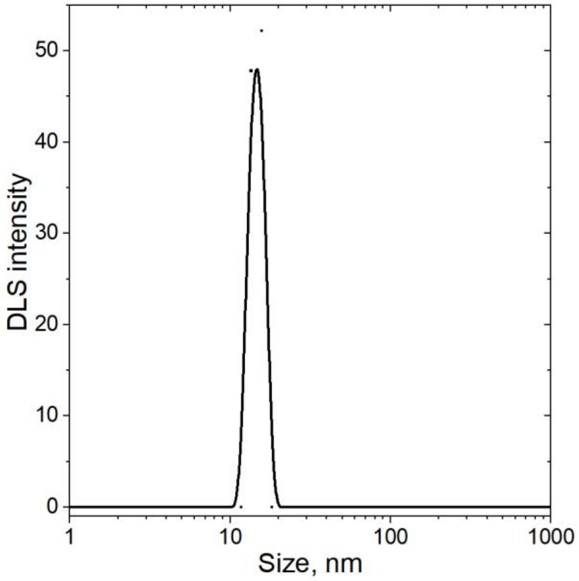

Figure S4. Size distribution of the CD-EDC/NHS conjugate determined by DLS.

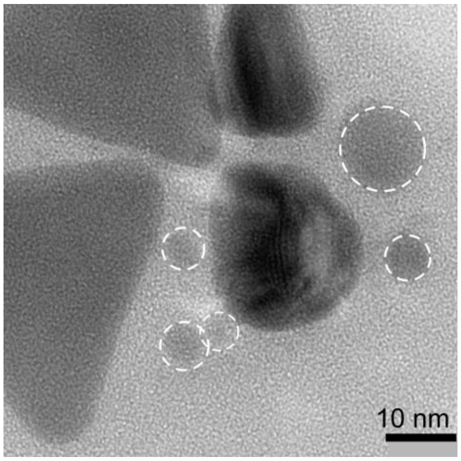

Figure S5. TEM image of CD-AgNP complex. CDs are highlighted by dashed circles.

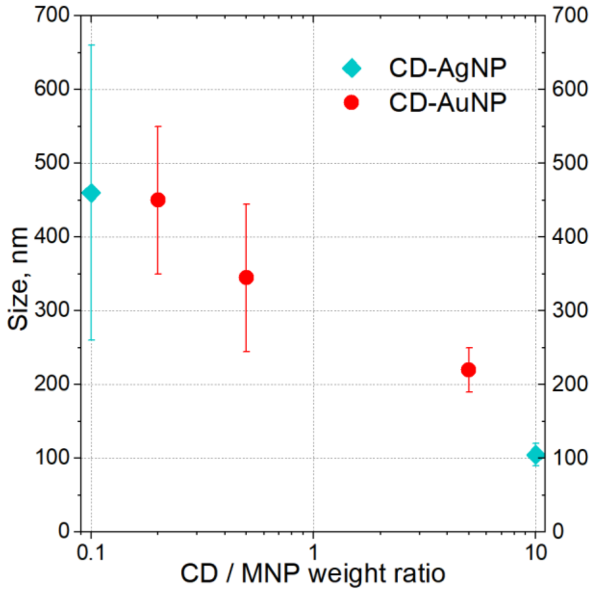

Figure S6. Hydrodynamic diameters of the complexes CD-AgNP (cyan diamonds) and CD-AuNP (red circles) as a function of the CD/MNP weight ratio.

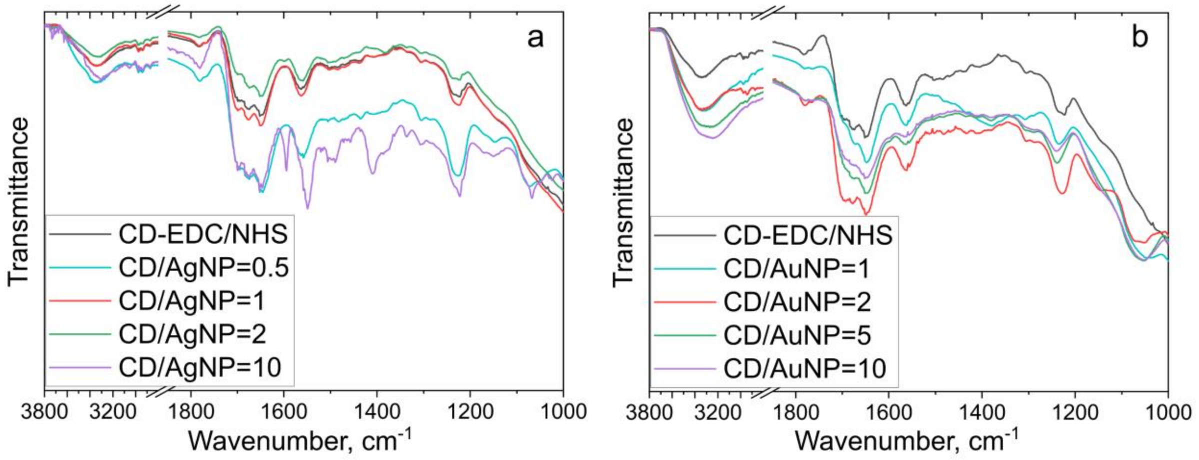

Figure S7. FTIR spectra of (a) CD-AgNP and (b) CD-AuNP complexes with different CD/MNP weight ratios (shown in different colors), compared with the one for the CD-EDC/NHS conjugate (shown in black).

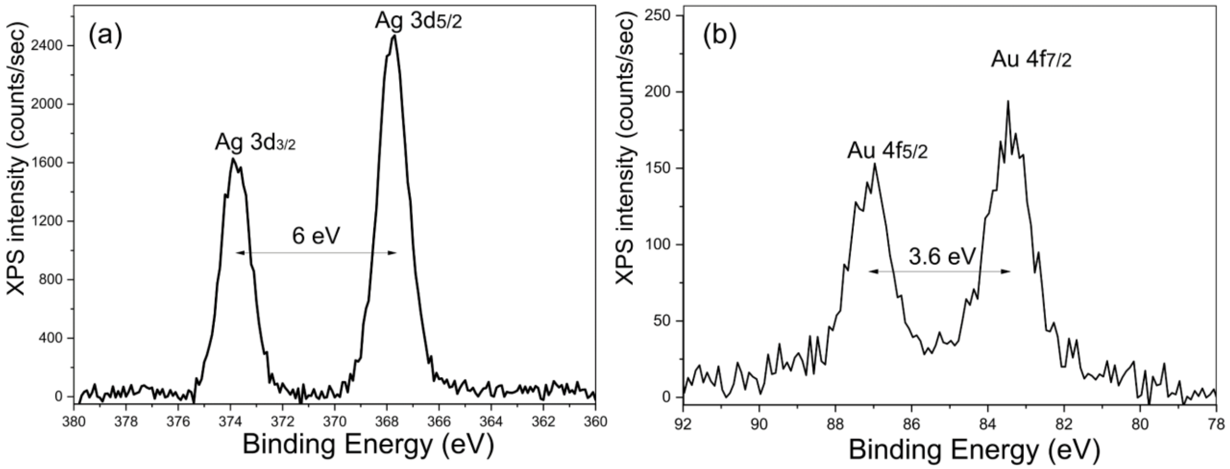

Figure S8. XPS spectra of (a) Ag binding energy in the CD-AgNP complex, and (b) Au binding energy in the CD-AuNP complex.

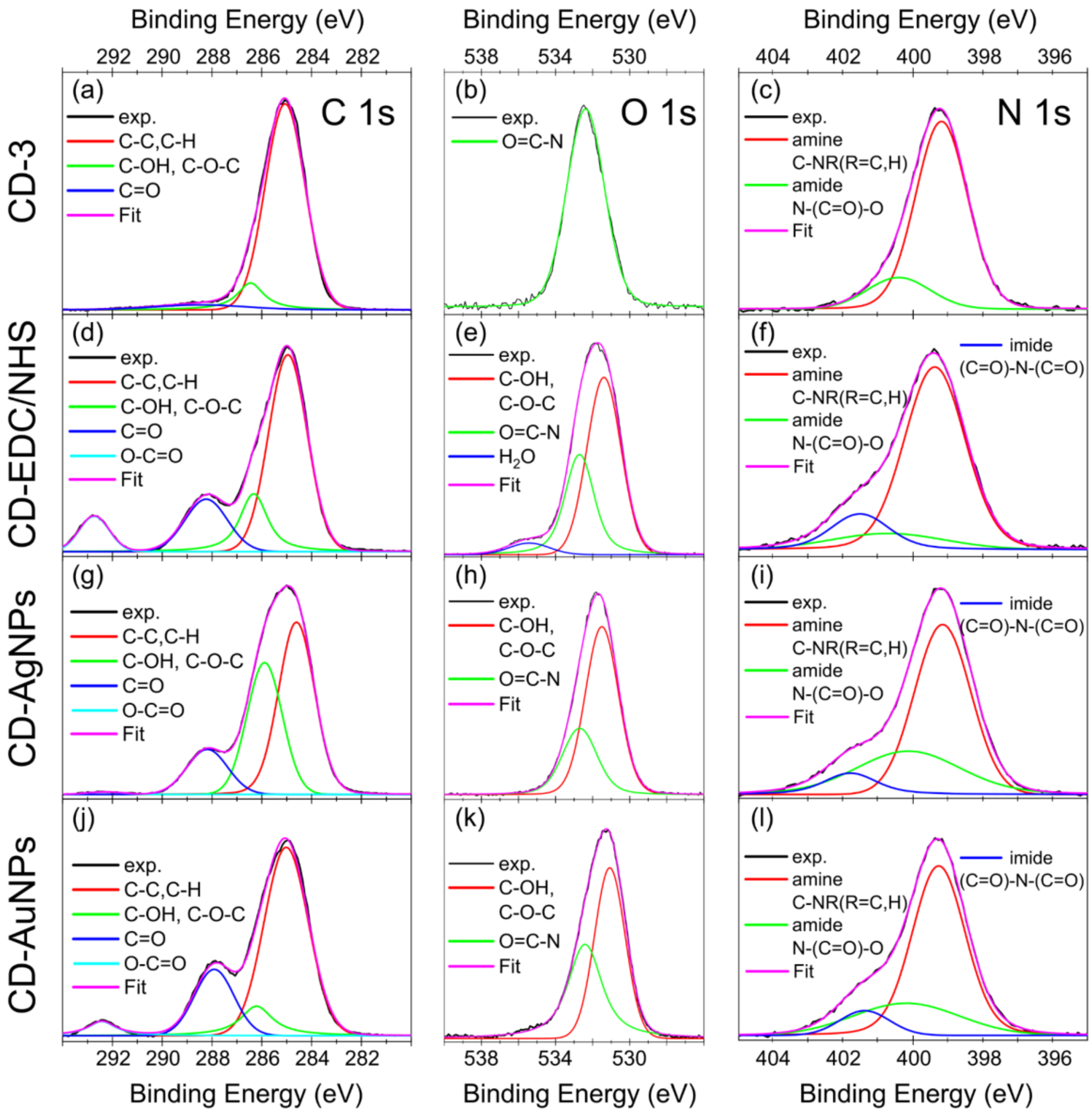

Figure S9. XPS spectra of C 1s (a, d, g, j), O 1s (b, e, h, k), and N 1s (c, f, i, l) of (a-c) CD-3, (d-f) CD-EDC/NHS conjugate, (g-i) CD-AgNP complex, and (j-l) CD-AuNP complex. Measured spectra are shown in black, their total fit – in pink, and their deconvolution into contributing peaks from different chemical bonds – in other colors as indicated on the frames.

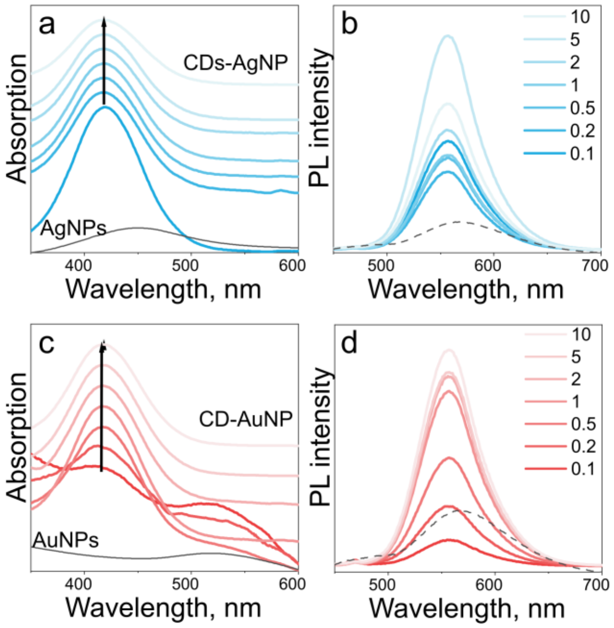

Figure S10. Optical properties of (a, b) CD-AgNP complexes and (c, d) CD-AuNP complexes. Absorption (a, c) and PL (b, d) spectra of the CD-MNP complexes with increasing CD/MNP weight ratio indicated by black arrows. Absorption spectra are vertically translated for clarity. Absorption spectra of the pristine AgNPs on the frame (a) and AuNPs on the frame (c) are shown by grey solid lines; PL spectrum of the pristine CD-3 in (b) and (d) is provided by grey dashed lines. Legend in (b) and (d) shows CD/MNP weight ratio.

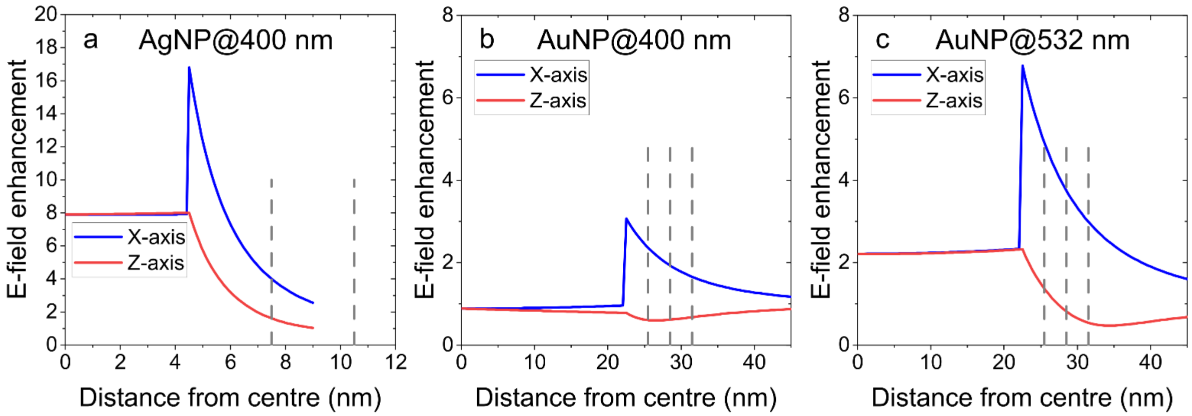

Figure S11. The E-field enhancement distribution along X-axis (blue) and Z-axis (orange) by 9 nm AgNP (a) and 45 nm AuNP(b,c) at 400 nm (a, b) and 532 nm (c). Dashed lines indicate the distances corresponding to 1, and 2 (3) CD diameters from the MNP surface.
